# Restricted human CD45 isoglycoforms serve as functional E-selectin ligands and delineate hematopoietic maturity

**DOI:** 10.1016/j.jbc.2025.110431

**Published:** 2025-07-01

**Authors:** Evan Ales, Robert Sackstein

**Affiliations:** Department of Translational Medicine and Translational Glycobiology Institute, Herbert Wertheim College of Medicine, Florida International University, Miami, Florida, USA

**Keywords:** CD45, CD45 isoform, isoglycoform, E-selectin ligand, sialyl Lewis X, translational glycobiology, sLeX, CD15s, E-selectin

## Abstract

CD45 is the most abundant glycoprotein on the surface of all nucleated hematopoietic-lineage cells, comprising multiple isoforms generated by alternative splicing of three exons (“A,” “B,” and “C”), which are exquisitely restricted across hematopoietic development. Despite the ubiquitous expression of CD45 on hematopoietic cells, its function(s) remain rather obscure. Here, we report that discrete CD45 isoforms expressed uniquely by immature human hematopoietic cells are distinguished as functional glycoforms (“isoglycoforms”) that bind E-selectin. Moreover, our studies indicate that “CD45RA,” a marker of human acute myeloid leukemia (AML) cells, identifies a distinct isoglycoform of CD45 containing all splice exon–encoded peptides. This isoglycoform, “CD45RABC-E,” is directly upregulated by AML cells and demarcates these malignant cells from mature human leukocytes and the native human hematopoietic stem cell. Further analyses revealed that treatment-resistant AML highly expresses CD45RABC-E. Our findings thus unveil heretofore unrecognized functions of CD45 and define a novel CD45 isoglycoform that delineates AML cells from life-sustaining human hematopoietic cells.

CD45 (protein tyrosine phosphatase receptor type C) is ubiquitously expressed on leukocytes and nucleated hematopoietic stem/progenitor cells. CD45 was initially characterized as a phosphatase with activity essential for T-cell receptor signal transduction in human T cells (1, 2). The CD45 glycoprotein consists of five unique isoforms created by combinations of three distinct, evolutionarily conserved, exon sequences denoted “A,” “B,” and “C” (Figs. S1 and S2) (3, 4). Expression of these exon-encoded peptides varies in a stage- and lineage-specific fashion, drawing attention to the functional implication of the peptides themselves. However, peptides serve as scaffolds for distinct glycan motifs, structures which, in themselves, confer unique biology. Thus, a full appreciation of any given glycoprotein’s biologic activity requires an understanding of the functional contribution(s) of its relevant glycosylations.

Glycans on glycoproteins are covalently linked to the core protein *via* nitrogen of asparagine (“N-linked”) or oxygen of serine or threonine (“O-linked”). Notably, the three exons generating CD45 isoforms encode peptides that harbor essentially all the O-linked glycans across the CD45 molecule (5, 6). This fact, coupled with the strict regulation of these isoforms, raises interest regarding the functional impact of isoform-specific glycosylations. Indeed, presently, the only molecules known to engage cell-surface CD45 are lectins (proteins that bind carbohydrate motifs): specifically, lymphocyte CD45 serves as a well-characterized ligand for the classes of lectins known as galectins and siglecs (7, 8, 9, 10, 11). In human T cells, CD45 isoforms are useful for distinguishing naïve *versus* memory cells, and lectin binding to the CD45 ectodomain is reported to induce CD45 phosphatase activity (12). However, conspicuously, among human nonlymphocytic leukocytes and human hematopoietic stem/progenitor cells (HSPCs), there is very limited understanding of the expression of specific CD45 isoforms, of their glycosylation profiles, and whether differential glycosylations on specific isoforms program lectin binding to CD45.

Monoclonal antibodies (mAbs) recognizing specific exon-encoded epitopes (*e.g.*, anti-CD45RA mAb) of human CD45 are well characterized (Fig. S2). Notably, anti-CD45RA mAbs, which recognize two CD45 isoforms (CD45RABC and CD45RAB), have utility in identifying acute myeloid leukemia (AML) cells and, possibly, leukemic stem cells (13, 14). However, importantly, human hematopoietic stem cells (HSCs) lack expression of “A” exon-encoded peptides (*i.e.*, human HSCs are “CD45RA negative”), and, thus, this immunophenotypic profile serves to differentiate AML cells from HSCs. To date, a biologic property related to the characteristic CD45RA positivity of AML cells has not been elucidated. However, variations in glycosylation patterns are implicated in the pathogenesis of AML (15) raising the possibility that glycan motifs displayed by certain CD45 isoforms could impact the pathobiology of this disease.

Over the past 2 decades, there has been an increasing interest in the role of E-selectin receptor–ligand interactions in both steady-state hematopoiesis and leukemogenesis (15, 16, 17, 18). E-selectin (CD62E) is an endothelial lectin (embedded in the name is “lectin,” and it is expressed uniquely on endothelial cells, thus, “E”-selectin). E-selectin binds to a tetrasaccharide motif known as sialyl Lewis X (sLeX; CD15s), which is presented by certain glycoproteins and glycolipids on immature and mature hematopoietic cell surfaces (collectively known as “E-selectin ligands”). E-selectin is expressed constitutively on specialized marrow microvessels and mediates cell–cell adhesive interactions that create the “vascular hematopoietic niche.” E-selectin is markedly upregulated in the marrow of patients with AML, and there is increasing evidence that E-selectin receptor–ligand interactions induce critical cell survival pathways that mediate AML treatment resistance (18). However, our knowledge into the identity of the E-selectin ligand(s) licensing this biology/pathobiology is incomplete.

In this study, we sought to uncover the breadth of E-selectin ligands expressed among human hematopoietic cells. Our results unveil the expression of heretofore unrecognized E-selectin-binding “isoglycoforms” of CD45 we collectively call “CD45E,” which are found exclusively on immature hematopoietic cells (*i.e.*, on human AML and healthy HSPCs but not on mature human leukocytes). Among AML cells, the sLeX-enriched CD45 isoglycoform containing all exon-encoded peptides (which we term “CD45RABC-E”) is preferentially expressed and upregulated. Collectively, our findings thus redefine the biology and function of CD45 and provide the first evidence that the CD45RABC-E isoglycoform of CD45 distinguishes human AML cells from life-sustaining multipotent HSPCs.

## Results

### Identification and biochemical characterization of “CD45RABC-E”

**Figure 1:**
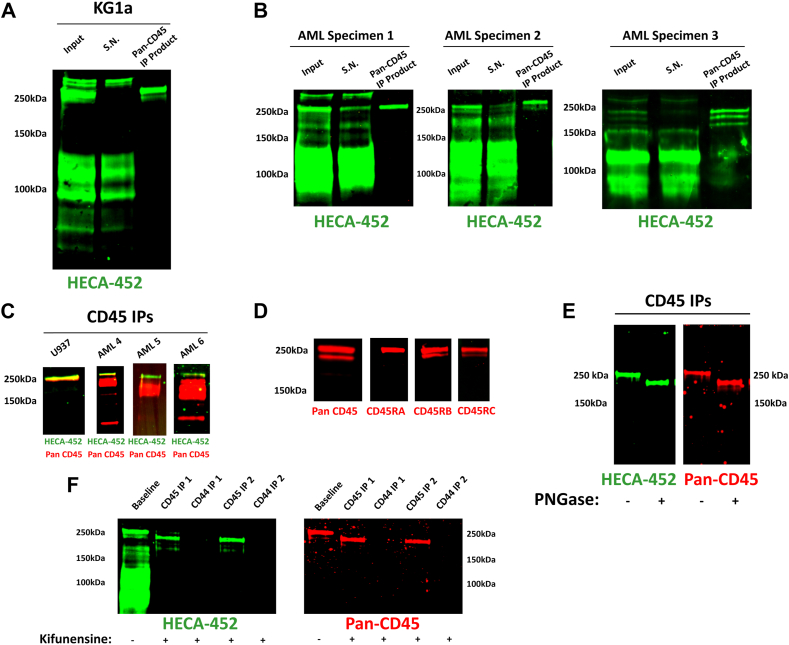
**“CD45RABC-E” is a novel and distinct E-selectin ligand displayed by human AML blasts.***A*, representative (n = 3) Pan-CD45 immunoprecipitation (IP) and subsequent Western blot staining with mAb HECA-452 mAb (to detect sLeX-decorated proteins) of lysates of KG1a cells. HECA-452 staining reveals that CD45, not CLA, is the ∼250 kDa E-selectin ligand routinely identified on Western blot analyses of HECA-stained KG1a lysates (S.N. = supernatant from the IP procedure [*i.e.*, nonimmunoprecipitated proteins]). *B*, Pan-CD45 and subsequent Western blot staining with mAb HECA-452 from lysates of three primary human AML specimens (from peripheral blood, >80% blasts) (S.N. = supernatant from the IP procedure [*i.e.*, nonimmunoprecipitated proteins]). *C*, utilizing LICOR dual-channel detection with HECA-452 mAb (*green*) and anti-CD45 mAb (*red*), CD45E was detected in U937 AML cell line and three primary human AML specimens (peripheral blood specimens; two with ∼40% blasts [specimens 4 and 5] one with ∼10% blasts [specimen 6]). *D*, Western blot staining of KG1a lysates utilizing all three exon-specific anti-CD45 mAbs confirmed that the E-selectin-binding glycoform of CD45 (“CD45E”) comprises two CD45 isoforms migrating at ∼250 kDa: predominantly CD45RABC (thus, CD45RABC-E), with minor component of CD45RBC (thus, CD45RBC-E). *E*, IP of CD45 (IP) from baseline and PNGase-treated KG1a lysates was performed with subsequent Western blot staining with HECA-452 mAb and pan-CD45 mAbs. Representative (n = 3) blot shown. *F*, IP of CD45 and CD44 (IP) from kifunensine-treated KG1a was performed with subsequent Western blot staining with HECA-452 and pan-CD45 mAbs. AML, acute myeloid leukemia; CLA, cutaneous lymphocyte antigen; mAb, monoclonal antibody; PNGase, peptide-*N*-glycosidase F; sLeX, sialyl Lewis X.

For several decades, a distinct high molecular weight (∼250 kDa) glycoprotein E-selectin ligand has been detected in Western blot analysis of human hematopoietic cells, and its identity was presumed to be “cutaneous lymphocyte antigen” (CLA) (16, 19, 20). CLA is a functional E-selectin-binding glycoform of PSGL-1 (CD162), a homodimeric molecule migrating at ∼250 kDa on SDS-PAGE, and, under reducing conditions, detectable by Western blot in its monomeric form (∼125–140 kDa). During this time, unpublished studies in our laboratory indicated that peripheral blood blasts from some patients with AML lacked expression of PSGL-1 as analyzed by flow cytometry, but Western blots of lysates of these blasts probed with either the anti-sLeX mAb HECA-452 or with E-selectin–Ig chimera (E–Ig) showed staining of a band migrating at ∼250 kDa. Other data from our laboratory indicated that CD45 immunoprecipitated from KG1a cells (a human CD34+ AML cell line that expresses the well-characterized glycoprotein E-selectin ligands HCELL, CD43-E, and CLA (16, 19, 20)) migrated on SDS-PAGE gel at this molecular weight. Accordingly, we stained Western blots of immunopurified CD45 from KG1a cells with HECA-452 mAb and found that it is the ∼250 kDa E-selectin ligand previously thought to be CLA (Fig. 1*A*). To determine whether expression of this novel E-selectin ligand is characteristic of primary human AML cells, we repeated these analyses with peripheral blood blasts of two primary human CD34+ AML specimens (AML specimens 1 and 2) and from a CD34-negative AML sample (AML specimen 3) with a high peripheral blood AML blast burden. We additionally attempted to detect this novel E-selectin ligand within total CD45 immunopurified from peripheral blood mononuclear cells (PBMCs) of patients with AML with varying levels of peripheral blood AML blast burden and, also, from the human CD34–negative AML cell line U937. Results of these studies provided further evidence that, in primary human AML cells, the high molecular weight E-selectin-binding glycoprotein long assumed to be solely comprised of homodimeric CLA, is, predominantly, CD45, and that expression of this novel E-selectin ligand is characteristic of human AML (Fig. 1, *B* and *C*).

To date, precise descriptions of CD45 isoforms have never been reported for myeloid cells, including AML blasts. We thus sought to define the CD45 isoforms of these myeloid-lineage cells and to define the isoform(s) capable of binding E-selectin as well as the particular exon-encoded CD45 peptide(s) that harbor sLeX motifs. To this end, we performed Western blot analysis with peptide-specific mAbs targeting each of the three peptides encoded by the alternatively spliced exons of CD45. Conspicuously, the isoform most consistently displaying E-selectin ligand activity on AML cells is CD45RABC, the largest and most glycosylated CD45 isoform containing all three alternatively spliced peptide sequences (Fig. 1*D*). These studies thus revealed that the highest molecular weight CD45 isoform is notably distinguished by sLeX decorations, thereby defining a unique glycoform (*i.e.*, an “isoglycoform”) we term “CD45RABC-E.”

All three splice exons of CD45RABC-E encode peptides located at the extracellular terminus of the glycoprotein that cumulatively display all the O-linked glycans present within the CD45 molecule (5, 6), implying that CD45RABC-E likely displays sLeX motifs on O-linked glycans. To directly elucidate the nature of the core carbohydrate linkages (*i.e.*, N- *versus* O-glycans) that display E-selectin-binding carbohydrates on CD45, we assessed the E-selectin binding capacity of CD45RABC-E after employing two well-validated techniques to trim/remove N-glycans from AML cells: enzymatic removal of N-glycans using peptide-*N*-glycosidase F (PNGase; New England Biolabs) and metabolic inhibition of N-glycosylation using kifunensine. In KG1a cells, use of each of these approaches led to removal of N-glycans, as evidenced by abrogation of sLeX display on HCELL (a CD44 glycoform that is a potent E-selectin ligand that displays sLeX only on N-linked glycans). However, conspicuously, neither PNGase nor kifunensine treatment abrogated CD45RABC-E’s E-selectin binding capacity (Fig. 1, *E* and *F*).

### Expression profile of “CD45E”

**Figure 2:**
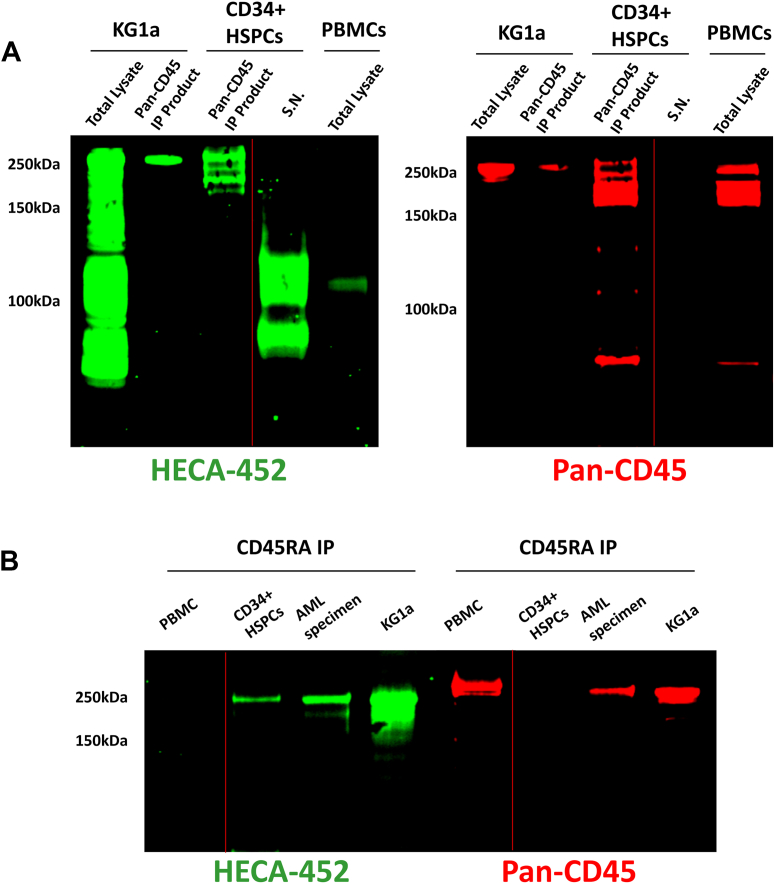
**CD34+ immature hematopoietic progenitors display numerous, discrete, CD45E isoglycoforms**. PBMCs lack expression of CD45E, and CD45RABC-E expression is prominent in AML. *A*, representative (n = 4) Western blot staining with HECA-452 mAb and with anti-pan CD45 mAb of total lysates of KG1a cells and primary healthy human PBMCs, of immunoprecipitation (IP) of CD45 from KG1a cells and from primary healthy human marrow HSPCs (CD34+ HSPCs from healthy donors), and of the IP supernatant (SN) of CD34+ HSPCs, in each case lysates equalized for equivalent input cell numbers. Multiple CD45E isoforms (beyond only CD45RABC-E) are expressed by CD34+ HSPCs, in contrast to the predominant CD45RABC-E species expressed by KG1a cells and the absence of CD45E expression in PBMCs. *B*, representative (n = 3) IP experiments utilizing anti-CD45RA mAb of equivalent amounts of lysates (*i.e.*, IP normalized for lysates equalized for input cell numbers) and subsequent Western blot staining with HECA-452 mAb and with anti-pan-CD45 mAb of primary healthy PBMCs, primary healthy human HSPCs (CD34+ HSPCs), a primary AML specimen, and KG1a cells (please note that Fig. S4*B* contains additional blots and the associated quantitative analysis). High-level expression of CD45E (dominantly as CD45RABC-E) is characteristic of AML, and PBMCs do not express any CD45E isoglycoforms. The *red line* indicates that the gel has been spliced to place lanes in continuity. AML, acute myeloid leukemia; HSPC, hematopoietic stem/progenitor cell; mAb, monoclonal antibody; PBMC, peripheral blood mononuclear cell.

Following the identification and characterization of CD45RABC-E expression among AML cells, we next investigated if native human hematopoietic progenitors could similarly display E-selectin-binding CD45 isoglycoform(s). Unlike AML, however, HSPCs are a collection of populations that include discrete subsets defined on the basis of CD45RA negativity. Though well established that human HSCs are CD45RA negative, no prior studies have defined the expression of CD45 isoforms among more mature human hematopoietic progenitors. Accordingly, to elucidate the CD45 isoforms, and, further, to assess if human hematopoietic progenitors use such isoforms to bind E-selectin, we performed immunoprecipitation (IP) and Western blot analyses of CD45 expressed on healthy CD34+ human HSPCs. Strikingly, we found that human HSPCs display multiple CD45 isoforms that bind E-selectin: CD45RABC, CD45RBC, and CD45RB (Fig. 2*A*). These results thus define a family of E-selectin ligands that we collectively call “CD45E.” Notably, multiple CD45E isoglycoforms were similarly present among PBMCs obtained from a patient diagnosed with a more mature (CD34-negative) AML subtype (AML specimen 3 in Figures 1*B* and S3).

As CD45RA positivity is thought to be specifically upregulated by AML cells and serves as a distinguishing biomarker of this disease, we assessed the relative expression of CD45RABC-E among HSPCs and AML. We therefore utilized an anti-CD45RA antibody to immunoprecipitate total CD45RABC from equivalent numbers of input AML cells and HSPCs and subsequently probed for E-selectin binding capacity. Consistently, we observed that human AML blasts display higher amounts of CD45RABC-E than do healthy CD34+ HSPCs (Figs. 2*B* and S4*B*). We quantitatively evaluated the relative expression of CD45RABC-E of CD34+ AML compared with that of CD34+ HSPCs by performing densitometry of HECA-452-stained Western blots of CD45RABC-E immunoprecipitants (immunopurified using anti-CD45RA) of five AML specimens compared with immunoprecipitants of three native bone marrow (BM) CD34+ HSPC specimens. Fold-change values of the densitometric levels of the HECA-452 staining of these blots were statistically analyzed by using a two-tailed *t* test, and this analysis indicated a significant difference between the densitometry-quantified expression of CD45RABC-E derived from CD34+ AML as compared with native CD34+ HSPCs (mean fold-change AML/HSPCs = 25.8; *p* < 0.01; Fig. S4*B*).

We additionally included anti-CD45RA immunoprecipitants derived from healthy PBMCs as a comparative control, and the data indicate that CD45E isoglycoforms containing the RA exon (*i.e.*, comprising CD45RABC-E) are characteristic of immature hematopoietic cells (AML and HSPCs) but are conspicuously absent among (mature) PBMCs. Thus, our data to date indicate that at least three different isoglycoforms of CD45E exist: CD45RABC-E, CD45RBC-E, and CD45RB-E.

**Figure 3:**
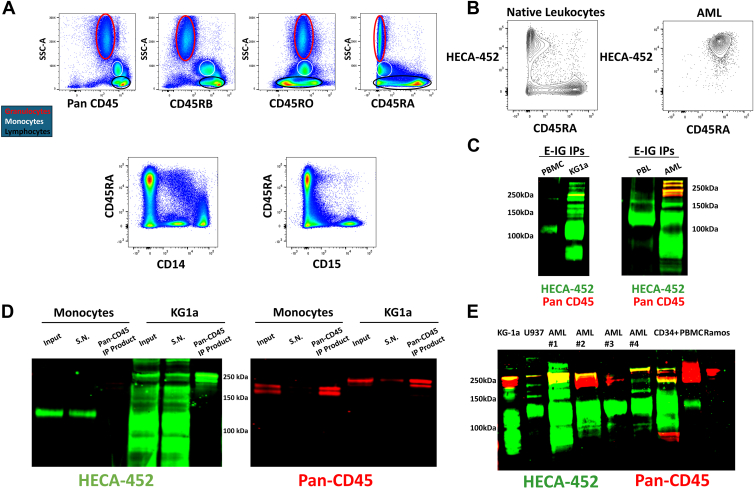
**Mature leukocytes do not express CD45E, but CD45E expression is characteristic of human AML cells.***A*, representative flow cytometry histogram of reactivity of total circulating cells to anti-CD45 mAbs recognizing, distinctly, Pan-CD45 (all isoforms), CD45RA-containing isoforms, CD45RB-containing isoforms, and the CD45RO isoform; in the top three panels, granulocytes (*red ellipse*), monocytes (*gray ellipse*), and lymphocytes (*black ellipse*) are each localized *via* their characteristic side scatter (SSC-A) profiles. In the bottom two panels, expression of CD45RA among monocytes and granulocytes was more specifically probed by staining cells with anti-CD14 mAb and anti-CD15 mAb, respectively. *B*, representative (n = 5) immunophenotyping with anti-CD45RA and HECA-452 (anti-sLeX) mAbs in native leukocytes and AML illustrates a mutual exclusivity of CD45RA/sLeX expression among native leukocytes and uniform convergence on primary human AML cells. *C*, IP with E–Ig and subsequent Western blot and LICOR dual-channel detection of staining with anti-pan CD45 mAb (*red*) and with HECA-452 mAb (*green*) from PBMCs (Ficoll-gradient interface cells; mononuclear leukocytes), PBLs (buffy coat cells obtained from healthy donors; “peripheral blood leukocytes,” total leukocytes), and KG1a/primary AML cells demonstrates that E–Ig fails to immunopurify CD45 from healthy PBMCs or PBLs, and CD45 protein from these cells does not react with HECA-452, in stark contrast to that of AML/KG1a cells. *D*, IP of CD45 derived from primary human monocytes and KG1a cells was performed with subsequent Western blot staining with HECA-452 mAb and anti-pan CD45 mAb. *E*, LICOR dual channel detection of a Western blot displaying staining with mAbs HECA-452 (*green*) and anti-pan CD45 (*red*) of AML cell lines and primary AML specimens, CD34+ HSPCs, mature leukocytes (PBMCs), and the Ramos cell line. AML, acute myeloid leukemia; E–Ig, E-selectin–Ig chimera; IP, immunoprecipitation; mAb, monoclonal antibody; PBMC, peripheral blood mononuclear cell; sLeX, sialyl Lewis X.

Given that our initial studies utilizing total lysate and anti-CD45RA immunoprecipitants derived from healthy PBMCs did not detect CD45E, we next sought to comprehensively investigate whether any CD45 isoform expressed by native human leukocytes also exists as “CD45E.” However, we have extremely limited knowledge regarding precise expression of CD45 isoforms among human leukocytes. Thus, to first gain needed insight into CD45 isoform expression of circulating, mature leukocytes, we probed these cells with mAbs directed toward the peptides encoded by the alternatively spliced exons of CD45 (Fig. 3*A*). These studies demonstrated that, in stark contrast to the immature myeloid cells of AML, circulating, mature myeloid cells are characteristically CD45RA negative. Monocytes coexpress predominantly CD45RO and CD45RB isoforms, whereas neutrophils largely express CD45RO alone. These results thus revealed that, strikingly, canonical E-selectin-binding leukocytes (*i.e.*, mature myeloid cells) are dominantly CD45RA negative. A significant percentage of circulating lymphocytes, however, are CD45RA positive (Fig. 3*A*).

Importantly, classical CD45RA-positive leukocytes, such as naïve T and B lymphocytes, predominately display the CD45RABC isoform and are known to be devoid of E-selectin ligands (10, 21, 22). This fact, combined with our data suggesting that mature human myeloid cells are CD45RA negative, highlights a critical distinction between native circulating human leukocytes and AML cells (*i.e.*, that CD45RABC-expressing mature leukocytes are devoid of E-selectin ligands, whereas AML cells utilize CD45RABC itself to bind E-selectin). To further explore this putative divergence between healthy leukocytes and AML cells, we performed comparative, biphenotypic analysis of CD45RA reactivity and E-selectin binding capacity between these cell types. Remarkably, CD45RA+ leukocytes indeed lack expression of E-selectin ligands, whereas AML blasts are commonly both CD45RA+ and bind E-selectin (Fig. 3, *B* and *C*). These findings underscore the absence of CD45RABC-E expression among mature leukocytes.

To more fully characterize the expression of CD45 isoglycoform(s) that could serve as E-selectin ligands among mature myeloid cells, we performed IP of CD45 from isolated blood monocytes, probing for E-selectin-binding capacity of the immunopurified CD45 (Fig. 3*D*). In a complementary approach, using an E–Ig as a probe, we immunoprecipitated all E-selectin ligands from AML cells, from PBMCs, and from peripheral blood leukocytes (obtained from buffy coat of whole blood cells), followed by CD45 staining Western blot analysis to detect if E-selectin-binding isoglycoforms of CD45 were present among these cells. While CD45 isoglycoforms that bind E-selectin were detected across all AML samples surveyed, CD45 derived from mature leukocytes was devoid of E-selectin binding (Fig. 3*C*).

These data highlight the ability of CD45E to delineate native and pathologic immature hematopoietic cells from mature leukocytes. This is especially true among AML cells as this CD45RABC-E isoglycoform seems to be preferentially expressed, and upregulated, in AML. This divergence can be observed in a Western blot of CD45E expression among various immature and mature hematopoietic cells and AML cells (Fig. 3*E*).

### Operational characteristics of CD45E

**Figure 4:**
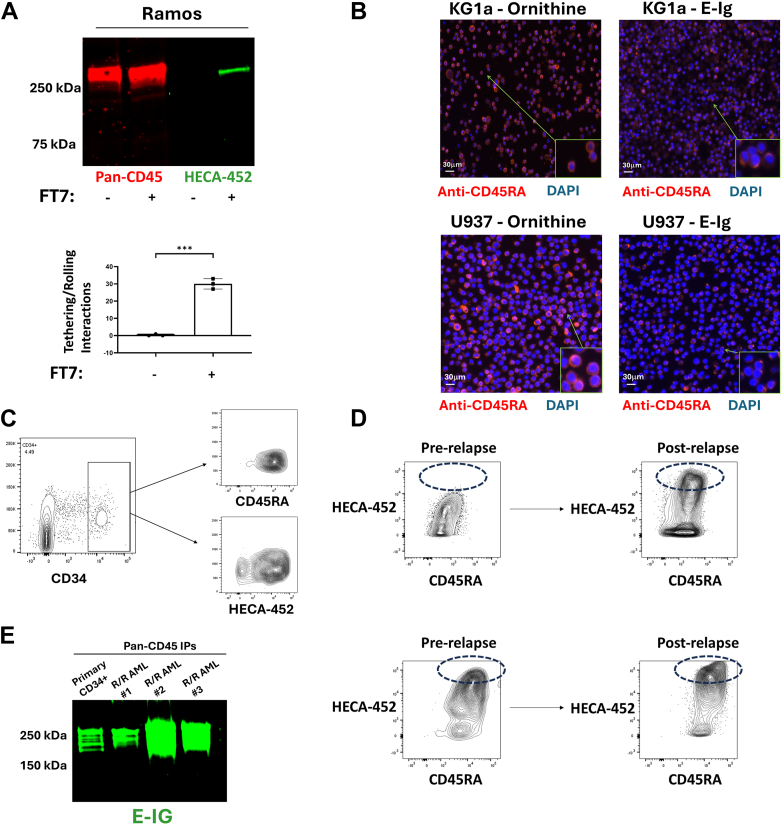
**CD45E is a functional E-selectin ligand and is highly expressed by treatment-resistant MRD and relapsed/refractory (R/R) AML cells.***A*, representative (n = 3) Western blot staining with HECA-452 (detecting E-selectin ligands) and pan-CD45 from lysates of exofucosylated (glycoengineered) Ramos cells and Raji cells (data not shown) illustrating the sole/predominant E-selectin ligand in the glycoengineered cells is “CD45RABC-E.” Quantification of E-selectin-mediated tethering and rolling interactions on TNF-activated HUVEC in the treated *versus* untreated Ramos cells as independent biologic replicates (n = 3). Values were compared with a two-tailed *t* test with each data point plotted with a bar representing the mean of these values with error bars displaying the associated standard deviation; ∗∗∗*p* < 0.0001. *B*, representative (n = 3, for each cell type) immunofluorescence (IF) microscopy image (staining with anti-CD45RA mAb [*red*] and DAPI [*blue*]) of KG1a (top two figures) and U937 cells (bottom two figures) adhered to ornithine (control, *left*) *versus* E–Ig (*right*). E–Ig binding induces capping of CD45. A *white scale bar* demonstrating approximately 30 μm is shown at the *bottom left* of each panel. *C*, representative (of n = 3) flow cytometry staining with anti-CD45RA mAb and HECA-452 of CD34+ cells isolated from bone marrow aspirates of patients with treated AML shows residual CD34+ cells are highly reactive with anti-CD45RA and HECA-452. *D*, immunophenotyping of two representative (n = 4) blood specimen from an R/R AML patient before and after treatment indicates that E-selectin-binding capacity is specifically increased among blasts surviving treatment Additionally, R/R AML blasts characteristically display high CD45RA positivity. *E*, immunoprecipitation (IP) and subsequent Western blot staining with E–Ig chimera of three patients with R/R AML, with comparison to primary healthy HSPCs (primary CD34+ marrow cells) shows that R/R AML displays high CD45E expression. AML, acute myeloid leukemia; DAPI, 4',6-diamidino-2-phenylindole; HSPC, hematopoietic stem/progenitor cell; HUVEC, human umbilical vein endothelial cell; mAb, monoclonal antibody; MRD, measurable residual disease; TNF, tumor necrosis factor.

After elucidating and elaborating the expression profile of the novel “CD45E” family, we aimed first to directly assess whether CD45E isoglycoforms can support physiologic E-selectin binding. To this end, we sought to determine whether CD45E, alone, could enable cell tethering/rolling on human umbilical vein endothelial cell (HUVEC). To avoid contribution(s) of non-CD45E E-selectin ligands, we utilized Ramos and Raji cells, which we determined can be modified by cell surface glycan engineering to display CD45 as their sole E-selectin ligand. Specifically, we observed that after treating Ramos and Raji cells with GDP-Fucose plus FTVII, an α(1,3)-fucosyltransferase (*i.e.*, after “exofucosylation”), CD45RABC-E serves as the sole sLeX-bearing, glycoprotein E-selectin ligand among the glycoengineered cells. Using a flow chamber device, we then introduced exofucosylated and native Ramos/Raji cells (*i.e.*, cells with or without CD45RABC-E, respectively) to HUVEC monolayers that had been tumor necrosis factor (TNF) activated to upregulate E-selectin expression. Analysis of E-selectin-mediated tethering/rolling interactions on the activated HUVEC monolayers under hemodynamic fluid shear conditions indicates that CD45RABC-E serves as a functional E-selectin ligand for both Ramos and Raji cells, and data for Ramos cells are shown in Figure 4*A*.

One of the additional, emerging operational features of E-selectin receptor–ligand interactions is the induction of cell survival pathway(s), and, specifically, its contribution to therapy resistance in hematological malignancies. Conspicuously, CD45 engagement of galectins on lymphocytes has been reported to induce cell signaling through “polarization” or “capping” of the surface CD45 glycoprotein (12). We thus next explored whether exposure of cells that express CD45RABC-E to E-selectin could induce capping of CD45RABC-E. Accordingly, we exposed KG1a and U937 cells to E-selectin. When engaging E-selectin, these cells underwent topographic polarization of the surface CD45RABC-E glycoprotein, indicating that E-selectin is indeed capable of functionally “capping” CD45RABC-E (Fig. 4*B*).

The observed E-selectin-induced capping of CD45E further raised the possibility that engagement of CD45RABC-E to E-selectin could be capable of supporting important cell survival pathways associated with the aggressivity/therapy resistance of AML cells. To explore this possibility, we first studied the disease-sustaining population of AML that comprises measurable residual disease (MRD). To assess the potential relevance of CD45RABC-E expression in these cells, we concurrently analyzed the presence of E-selectin ligands and CD45 isoform display within human MRD samples (n = 3) derived from BM aspirates of patients with AML. Importantly, we observed that MRD AML cells characteristically display high E-selectin-binding capacity and are largely marked by CD45RA positivity (Fig. 4*C*). Further immunophenotyping of these CD34+ MRD specimens indicated low expression or the absence of CD38 expression, which is consistent with an immature, leukemia-regenerating cell phenotype.

The natural progression of MRD is the development of treatment-resistant AML (relapsed/refractory [R/R] AML). Importantly, treatment-resistant AML cells reportedly contain higher total E-selectin-binding capacity (15, 23). To assess if a similar pathobiology would be observed within a given patient treatment course, we compared the average E-selectin-binding capacity of AML cells before and after relapse and/or treatment resistance within a given patient. We additionally analyzed the expression of CD45 isoforms within these AML cells to elucidate the potential expression of CD45E. Strikingly, the E-selectin-binding capacity of AML cells was higher after relapse or with treatment resistance, and, notably, high levels of CD45RA positivity were retained at all time points measured (n = 4; Figs. 4*D* and S5). Next, to directly assess the expression of CD45E in R/R AML, we immunoprecipitated all CD45 isoforms from R/R AML specimens and found that these treatment-resistant cells express high levels of CD45E (Fig. 4*E*).

## Discussion

E-selectin receptor/ligand interactions have well-recognized roles in many aspects of hematopoiesis/leukemogenesis (18). Though it had been presumed that all glycoprotein E-selectin ligands expressed on human hematopoietic-lineage cells were already characterized, our studies here reveal that an E-selectin ligand previously perceived to be CLA (PSGL-1) is CD45, specifically, an sLeX-decorated isoglycoform of the largest isoform of CD45, a molecule we now designate “CD45RABC-E.” The discovery of this novel, functional E-selectin ligand and the results of our studies into its structure, expression, and operational capacities offer unique insights into CD45 biology.

Although CD45 is a ubiquitous and well-known marker of hematopoietic cells, its structural features are relatively uncharacterized, and even less is known about its function for most cell subsets. Prior studies have indicated the importance of CD45’s phosphatase activity within both HSPCs and AML (24, 25, 26, 27), but no prior studies have provided information on whether any CD45 isoforms are specialized to engage in cell–cell interactions. CD45RA positivity as quantified by flow cytometry is a well-characterized marker of AML cells, including the leukemic stem cell, and conversely, CD45RA negativity is part of the current and conventional immunophenotype of human HSCs (13, 14); still, the precise isoform(s) being identified with anti-CD45RA mAbs in these studies, and their function(s), have not been reported previously.

In this study, we have identified the full spectrum of isoglycoforms of CD45 that serve as E-selectin ligands (collectively, “CD45E” molecules). We draw attention to the fact that despite clear evidence of cell surface CD45 expression by flow cytometry, we have observed that manipulations of cell lysates for extended periods above freezing temperatures can affect integrity of CD45. This observation may account for the apparent inability to detect any CD45E molecule in our prior report on the E-selectin ligands expressed on CD34+ HSPCs, whereby lysates were exhaustively precleared of other, previously identified glycoprotein E-selectin ligands (*i.e.*, HCELL, CD43-E, and CLA), and there were no residual bands detected by Western blot staining using either E–Ig or mAb HECA-452 (19). Here, we describe distinct isoglycoforms of CD45 that function as E-selectin ligands on healthy human hematopoietic progenitor cells and on AML blasts, providing functional insights into CD45 structural biology that greatly extend our understanding regarding this definitional and ubiquitous cell surface marker of hematopoietic-lineage cells.

The results herein thus expand our understanding of E-selectin receptor–ligand interactions and CD45 function by establishing that AML preferentially expresses, and upregulates, a unique and functional E-selectin-binding isoglycoform of CD45, CD45RABC-E. Our data indicate that this isoglycoform is structurally licensed to enable potent E-selectin binding as it contains peptides encoded by all three alternatively spliced exons, each of which displays O-linked carbohydrates carrying E-selectin-binding motifs. These data represent the first assignment of a precise CD45 isoglycoform to a human myeloid cell and provide insights into its role in supporting the lodgment of AML cells and HSPCs within marrow vascular hematopoietic niches.

By clarifying the structural biology of CD45 among hematopoietic cells, our data further indicate that multiple E-selectin-binding CD45E isoglycoforms can exist (*e.g.*, CD45RABC-E, CD45RBC-E, and CD45RB-E) but are each exclusively restricted to immature hematopoietic cells (*i.e.*, CD34+ HSPCs and AML blasts). Conspicuously, a CD34-negative AML sample displayed multiple CD45E isoglycoforms (CD45RABC-E, CD45RBC-E, and CD45RB-E), indicating a shift in CD45 isoforms commensurate with its maturation (*i.e.*, shifting away from CD45RABC-E as occurs across differentiation of oligopotent progenitors to more mature myeloid leukocytes). These data highlight the complex regulation of the CD45 glycoprotein among the hierarchical differentiation of hematopoietic cells. Strikingly, no mature subset of human leukocytes displays any CD45E species, even those expressing CD45RABC or high levels of other glycoprotein E-selectin ligands. Our findings additionally reveal an immunophenotypic dichotomy wherein naturally occurring mature leukocytes displaying E-selectin ligands are characteristically CD45RA negative (including mature myeloid cells), thereby indicating that this expression profile can serve to delineate mature from immature native and malignant hematopoietic cells. In summary, we find that CD45RABC-E, CD45RBC-E, and CD45RB-E can all exhibit E-selectin-binding capacity (*i.e.*, excluding CD45RAB [a relatively minor isoform] and CD45RO [the isoform with none of the alternatively spliced exons that encode sequences that carry all the O-glycans across the molecule]).

To determine whether sLeX-decorated isoglycoforms of CD45 serve as functional E-selectin ligands, we utilized a highly precise glycoengineering method (*i.e.*, exofucosylation) to solely enforce CD45E expression on Raji and Ramos cells. This approach allowed for the assessment of the ability of CD45E, alone, to mediate cell tethering and rolling on endothelial cells expressing E-selectin, and the results provided clear evidence that CD45E exhibits functional E-selectin binding capacity under physiologic fluid shear conditions. Though E-selectin is a well-recognized molecular effector of adhesive interactions creating marrow hematopoietic growth “niches,” the identity(-ies) of the distinct E-selectin ligand(s) that mediate the cell signaling pathway(s) promoting hematopoietic progenitor cell proliferation and survival are still unknown. Notably, previous studies have suggested that total E-selectin ligand expression is higher in treatment-resistant AML and that chemotherapy may directly drive AML blast sLeX expression (15, 23, 28). In agreement with these findings, our data suggest that E-selectin binding capacity is markedly increased in R/R AML and that these treatment-resistant cells highly express CD45E. Future studies should be directed to determine how CD45E can directly influence specific, E-selectin-induced, cell signaling pathways.

Our study has identified distinct isoglycoforms of CD45 that function as E-selectin ligands on healthy human hematopoietic progenitor cells and on AML blasts, providing functional insights into CD45 structural biology that greatly extend our understanding regarding this definitional and ubiquitous cell surface marker of hematopoietic-lineage cells. Our data reconcile prior reports of aberrant CD45RA positivity and high E-selectin-binding capacity in AML by elucidating that CD45RABC-E itself binds E-selectin. Our findings thereby raise the potential for specifically targeting the CD45RABC-E isoglycoform of CD45 in therapy of AML. Considering the feasibility of this approach, it is notable that CD45 is a potent immunogen as evidenced by the number, fidelity, and multispecies origins of existing anti-CD45 mAbs, and, in particular, anti-CD45 mAbs have been raised that recognize glycosylation-dependent CD45 epitopes (*e.g.*, MEM55 is reported to be absolutely dependent upon the O-linked glycosylation encoded by the alternatively spliced exons of CD45) (10, 22). In addition, anti-CD45 mAbs (*e.g.*, ^90^Y-conjugated-anti-CD45 mAbs (29)) are already being employed for purging marrow hematopoietic cells in preparative regimens for HSC transplants. Accordingly, further studies are warranted to gain greater knowledge of the distinguishing proteoglycomic features of CD45RABC-E, information which will be key to the development of novel diagnostic, prognostic, and therapeutic approaches for AML based on the expression of this previously unrecognized human CD45 isoglycoform.

## Experimental procedures

### Cell culture

Human leukemia/lymphoma cell lines (KG1a, Ramos, Raji, and U937) were purchased from the American Type Culture Collection. Cells were maintained as reported (16) and routinely tested for the absence of Mycoplasma contamination.

### Patient specimen processing

All human cells were obtained and used in accordance with the procedures approved by the Human Experimentation and Ethics Committees of Massachusetts General Brigham Healthcare and Dana-Farber Cancer Institute and the Institutional Review Board of the Florida International University. These human studies abide by the Declaration of Helsinki principles. All human AML samples were defined by blast expression of CD34 and/or side scatter properties. Human PBMCs were processed as previously described (30). Healthy HSPCs were isolated from discarded filter sets of BM harvests performed for clinical HSC transplantation. BM mononuclear cells were isolated by Ficoll–Hypaque density separation, and human cells enriched for HSPCs (CD34+ cells) were collected by positive selection using anti-CD34 immunomagnetic beads (StemCell Technologies). AML primary specimens utilized for Western blot analysis contained >70% CD34+ blasts within the mononuclear fraction as confirmed by flow cytometry for each sample unless otherwise noted.

### Flow cytometry

For immunophenotyping, cells were processed and stained as previously described (21). Antibodies utilized included HECA452-FITC (catalog no.: 321308; BioLegend), HECA452-BV421 (catalog no.: 563961; Becton–Dickinson [BD]) Pan-CD45-BUV496 (clone HI130, catalog no.: 563961; BD), anti-CD45RA-PE (clone HI100, catalog no.: 555489; BD), anti-CD45RB-BV421 (clone MT4, catalog no.: 744902; BD), anti-CD45RC-AF647 (clone MT2, catalog no.: 565857; BD), anti-CD38-BV605 (catalog no.: 356642; BioLegend), anti-CD34-APC/Cy7 (catalog no.: 343514; BD), and anti-CD123-PE/Cy7 (catalog no.: 560826; BD). For E–Ig flow cytometry—mouse E-selectin–Ig chimera (catalog no.: 575-ES; R&D Systems), rabbit anti-6-His-FITC (catalog no.: A190-114F; Bethyl). Cytometry was performed on a BD A3 cytometer, then data were exported and analyzed on FlowJo (BD). Single-color controls were included to perform compensation, which was completed on FlowJo.

### Western blot analysis

Western blots were performed as previously described and validated (19). Blots were stained with mAb HECA-452 (BioLegend) or the following antihuman mAbs: pan-CD45 (catalog no.: D9M8I; Cell Signaling), CD45RA (BioLegend), CD45RB (BioLegend), and CD45RC (BioLegend). Importantly, the anti-CD45 mAb (catalog no.: D9M8I) utilized throughout the Western blots and IPs was targeted toward the intracellular domain of CD45 and is isoform/glycoform independent. Murine E-selectin–human Fc chimera (E–Ig) was from Biotechne, and E–Ig staining was performed as previously described (19). Stained membranes were visualized and analyzed with the Li-Cor Odyssey imager utilizing pertinent Li-Cor fluorescent antibodies. Membrane analysis, signal quantification, and data export were performed using Image Studio Lite software (Li-Cor). GraphPad Prism (GraphPad Software, Inc) was used for statistical analyses of quantified signal intensities. Signals were compared using one-way ANOVA. All Western blot lanes were normalized in each lane by an equivalent cell number.

### Immunoprecipitation

IP with either mAbs or E–Ig was performed as previously described (19), and Western blot analysis was performed as described previously. Western blots containing an IP were normalized across each lane to load an equivalent cell number for each specimen.

### Immunofluorescence

Ornithine or E–Ig was coated to plastic cell culture plates overnight at 4 °C. KG1a or U937 were subsequently adhered to coated plates under rotational shear stress (60 RPM). After 30 min, unadhered cells were washed with PBS, and adhered cells were fixed with 4% formaldehyde for 10 to 30 min at room temperature. Cells were stained with AF647-conjugated anti-CD45RA (BioLegend) and 4',6-diamidino-2-phenylindole (BioLegend) according to the manufacturer’s recommendations, and fluorescence was visualized with EVOS FL Imaging System (Life Technologies). Anti-CD45RA mAb specificity for immunofluorescence was validated by staining a positive (CD45RA+) and negative (CD45RA-) control (KG1a and HL-60, respectively).

### Parallel plate flow chamber assays

Parallel plate flow chamber assays were performed as previously described (30). Briefly, HUVECs were grown to confluence to mimic human endothelium on a BioFlux plate specifically designed to generate and visualize physiologic shear-stress conditions (BioFlux 200+). Once grown to confluence on the BioFlux plate, HUVEC cells were stimulated with TNF-alpha for 4 h to induce expression of E-selectin. After 4 h of TNF stimulation, cells of interest were introduced to the activated HUVEC monolayer under defined, physiologic shear-stress ranges (2–4 dyn/cm^2^). Shear-resistant, E-selectin-dependent interactions were visualized with the EVOS imaging system, and differences among groups were quantified by Prism. Values were compared with a two-tailed *t* test.

### Modification of cell surface glycans

To enzymatically cleave N-glycans, cell lysates were treated with PNGase-F according to the manufacturer’s instructions. For metabolic inhibition of N-glycosylation, cells were incubated with 1 μg/ml kifunensine (Cayman chemicals) for 4 days. Exofucosylation to enforce sLeX expression was achieved by treating cells with α(1,3)-fucosyltransferase FTVII as previously reported (30).

## Data availability

The data underlying this manuscript are present in the main article and supporting figures.

## Supporting information

This article contains supporting information including supporting figures.

## Conflict of interest

The authors declare that they have no conflicts of interest with the contents of this article.
