# Supplementary material for: Restricted human CD45 isoglycoforms serve as functional E-selectin ligands and delineate hematopoietic maturity
Source: J Biol Chem. 2025 Jul 1;301(8):110431. doi: 10.1016/j.jbc.2025.110431 (PMC12336693; doi:10.1016/j.jbc.2025.110431)
Supplement: Supplemental Figures [file mmc1.docx]

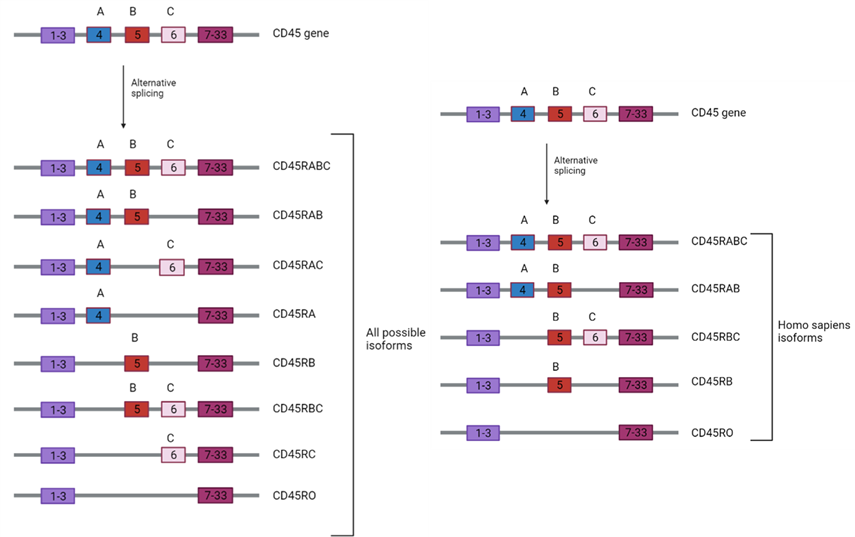


**Supplemental Figure 1. Alternative splicing of the *PTPRC* gene (that encodes the CD45 protein).** Three consecutive exons (3, 4, 5; termed A, B, and C, respectively) of the *PTPRC* gene are alternatively spliced to create eight possible isoforms. Substantial amounts of only five of these have been isolated as cDNA and identified at the protein level within the human, whereas all eight have been identified in the rat and mouse.


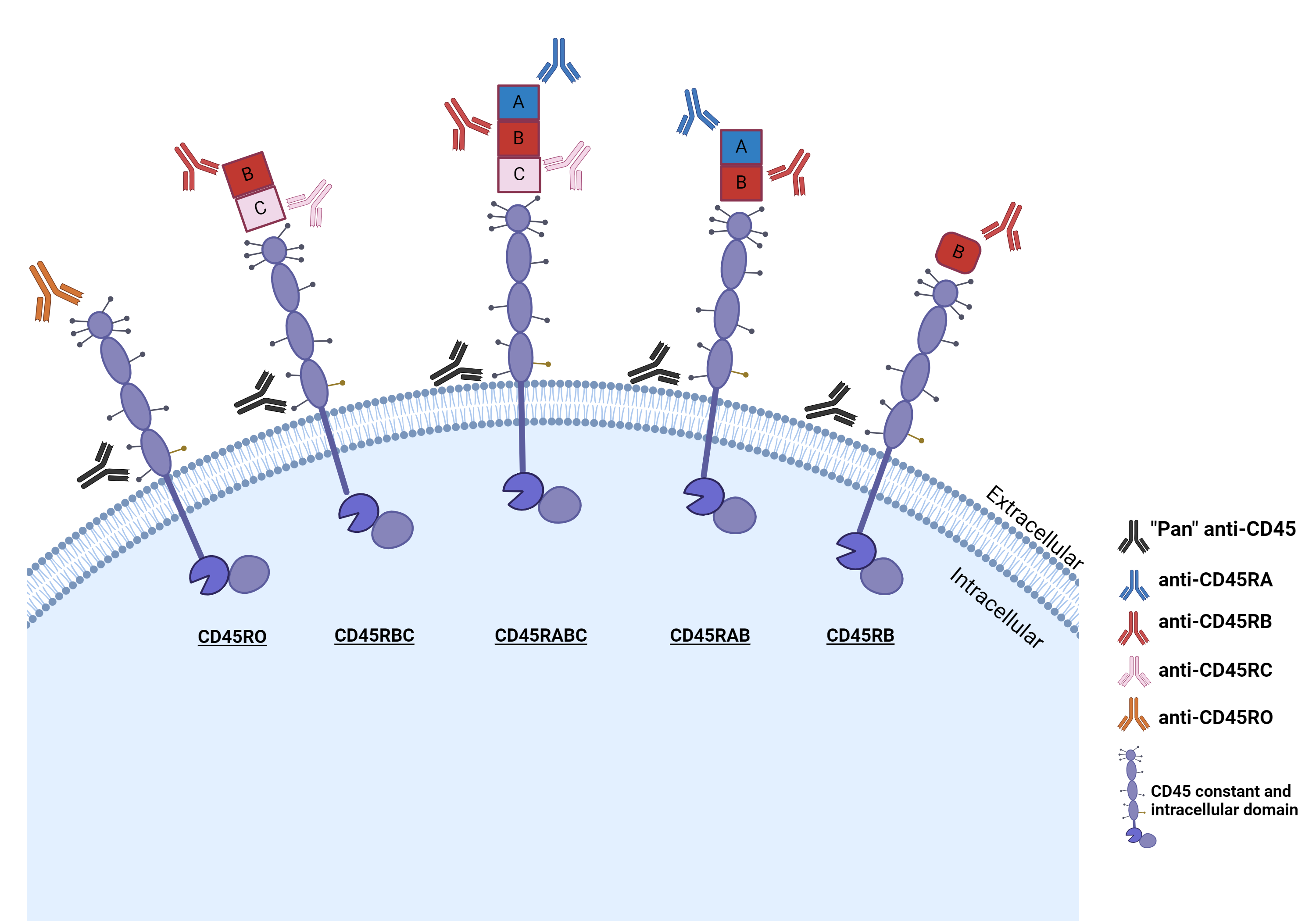


**Supplemental Figure 2. “Isoform-specific” anti-CD45 mAbs recognize multiple CD45 isoforms.** mAbs utilized to identify CD45 isoforms are directed towards peptide sequences encoded by one specific alternatively spliced exon. For instance, anti-CD45RA mAbs recognize peptides encoded by the “A” exon and thus identify either CD45RABC or CD45RAB in humans. Anti-CD45RB mAbs will recognize all isoforms containing alternatively spliced exons, (i.e., all CD45 human isoforms except CD45RO contain a CD45RB exon and thus react with anti-CD45RB mAbs). Conversely, the anti-CD45RO mAb recognizes only the CD45RO isoform.


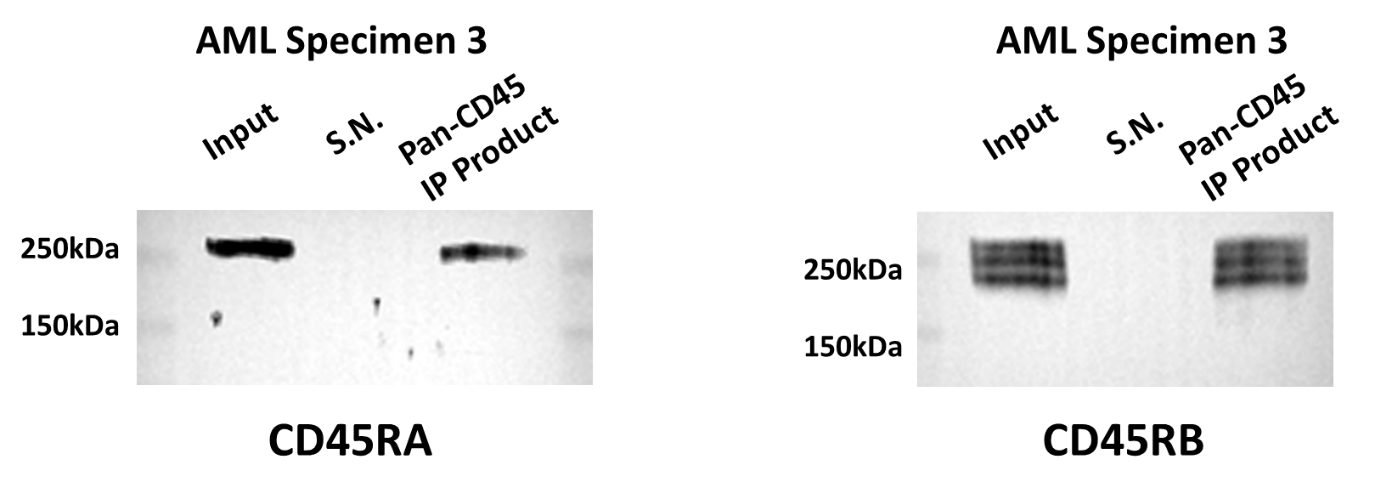


**Supplemental Figure 3.** AML specimen 3 Pan-CD45 immunoprecipitation (IP) and subsequent western blot staining with either anti-CD45RA or anti-CD45RB (S.N. = supernatant from the immunoprecipitation procedure (i.e., non-immunoprecipitated proteins)). The data show that three CD45E isoglycoforms exist in this less mature (CD34-) AML specimen: CD45RABC-E, CD45RBC-E, and CD45RB-E.


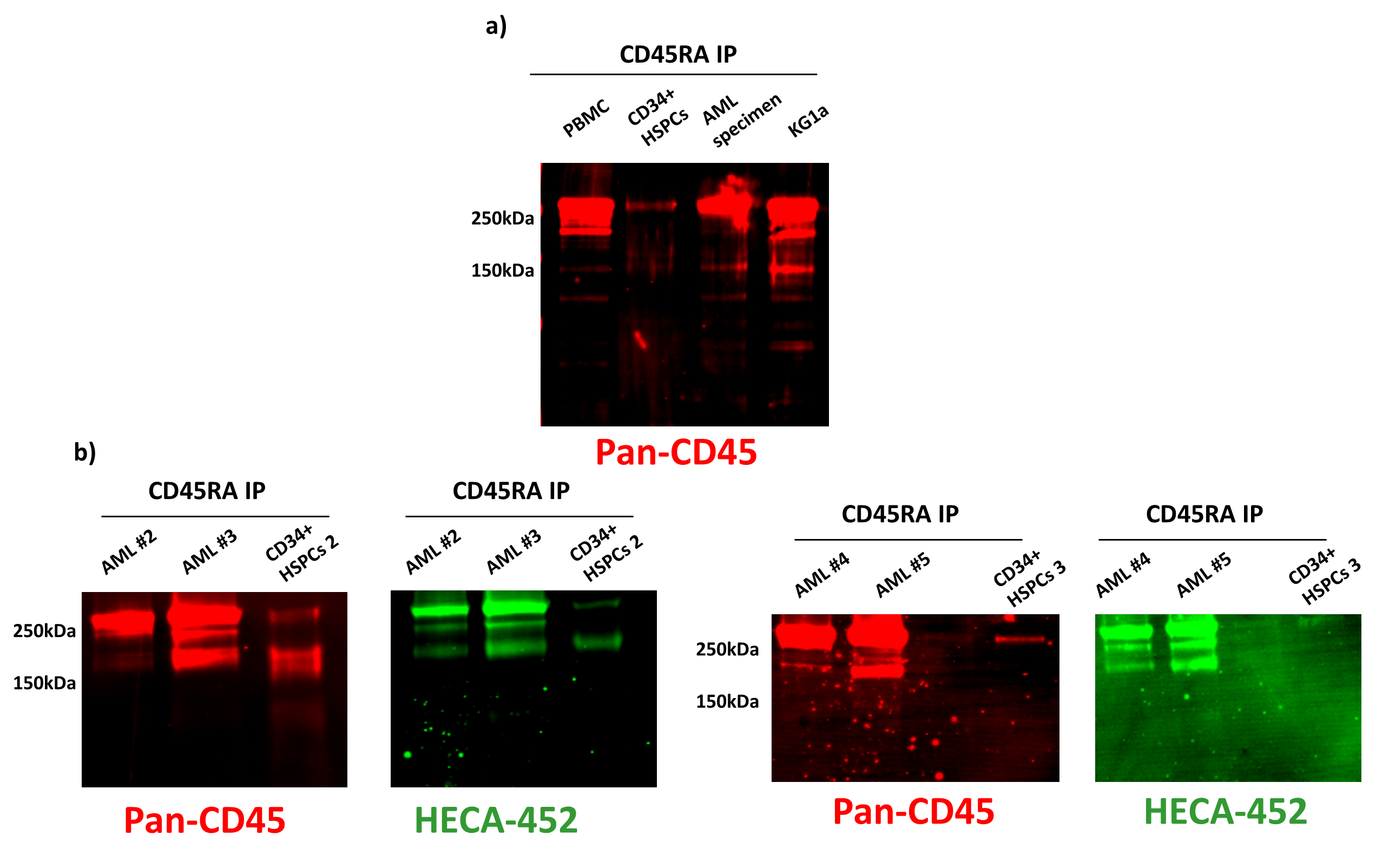


**Supplemental Figure 4. (a)** *Higher exposure of the same blot from Figure 2b.* Immunoprecipitation (IP) of equivalent amounts of lysates with anti-CD45RA mAb and higher exposure of western blot staining with pan-CD45 of primary healthy PBMCs, primary healthy human HSPCs (CD34+ HSPCs), a primary AML specimen, and KG1a cells, and shows a high level expression of CD45E (as CD45RABC-E) in AML and the presence of CD45RABC but *not* CD45RABC-E among PBMCs. **(b)** Two replicated experiments from Figure 2b performing immunoprecipitation (IP) of equivalent amounts of lysates with anti-CD45RA mAb and subsequent western blot staining with HECA-452 mAb of two separate primary human CD34+ HSPC collections (CD34+ HSPCs) and four primary AML specimens shows a high-level expression of CD45E (as CD45RABC-E) in AML and a relatively minor level of expression by CD34+ HSPCs. As mentioned in the text, we further evaluated the relative expression of CD45RABC-E of HECA-452-stained western blots of CD34+ AML compared to that of CD34+ HSPCs by performing densitometry of CD45RABC-E immunoprecipitants of all five AML specimens utilized compared to that of the three native bone marrow CD34+ HSPC specimens utilized. Fold-change values of the densitometric levels of HECA-452-staining of these blots were statistically analyzed by using a two-tailed t test and this analysis indicated a significant difference between the densitometry-quantified expression of CD45RABC-E from CD34+ AML as compared to native CD34+ HSPCs (mean fold-change AML/HSPC = 25.8; P<0.01).


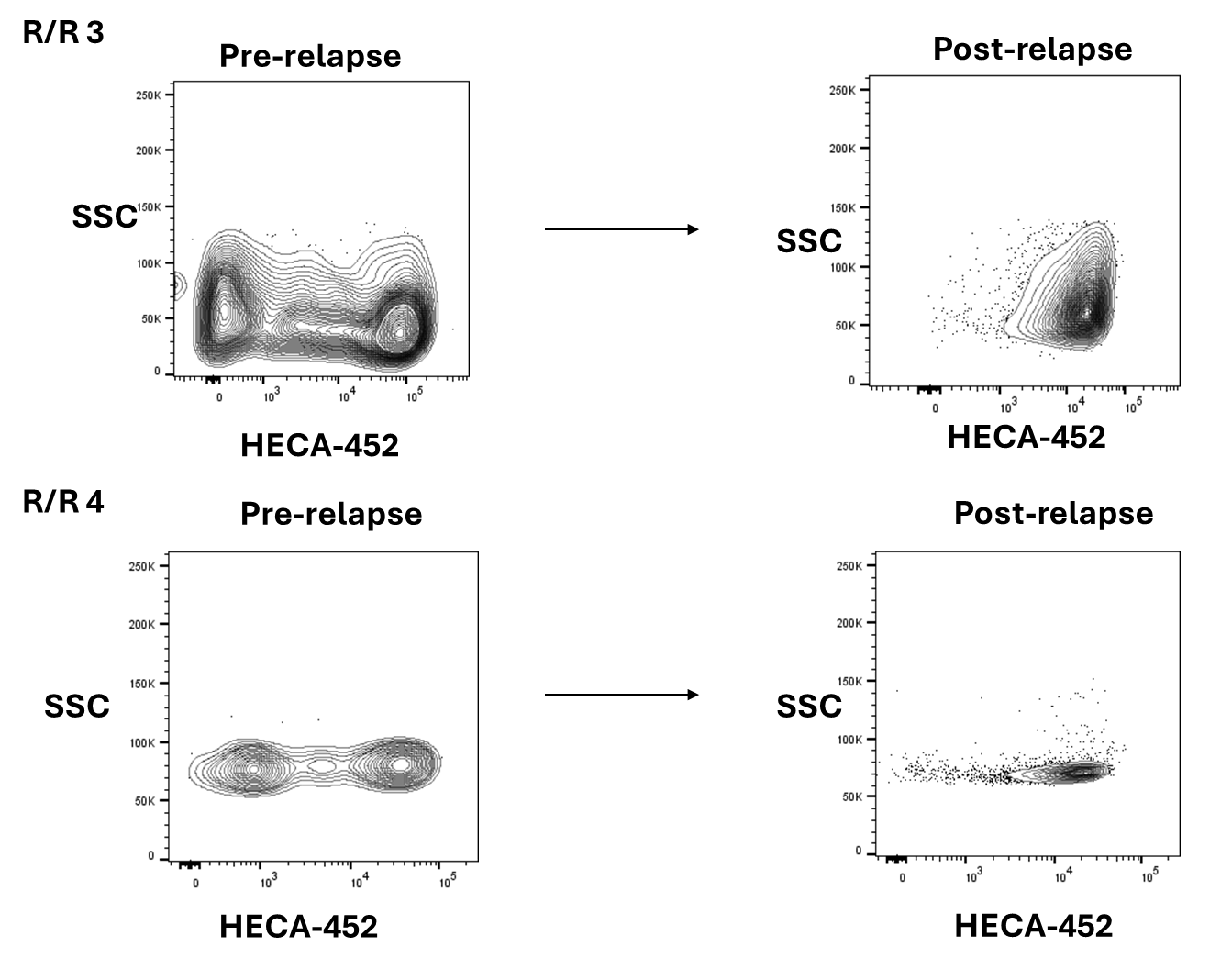


**Supplemental Figure 5.** Immunophenotyping of separate specimens of Relapsed/Refractory (R/R) AML blasts from patients before and after treatment indicates that E-selectin-binding capacity is specifically increased among blasts surviving treatment.


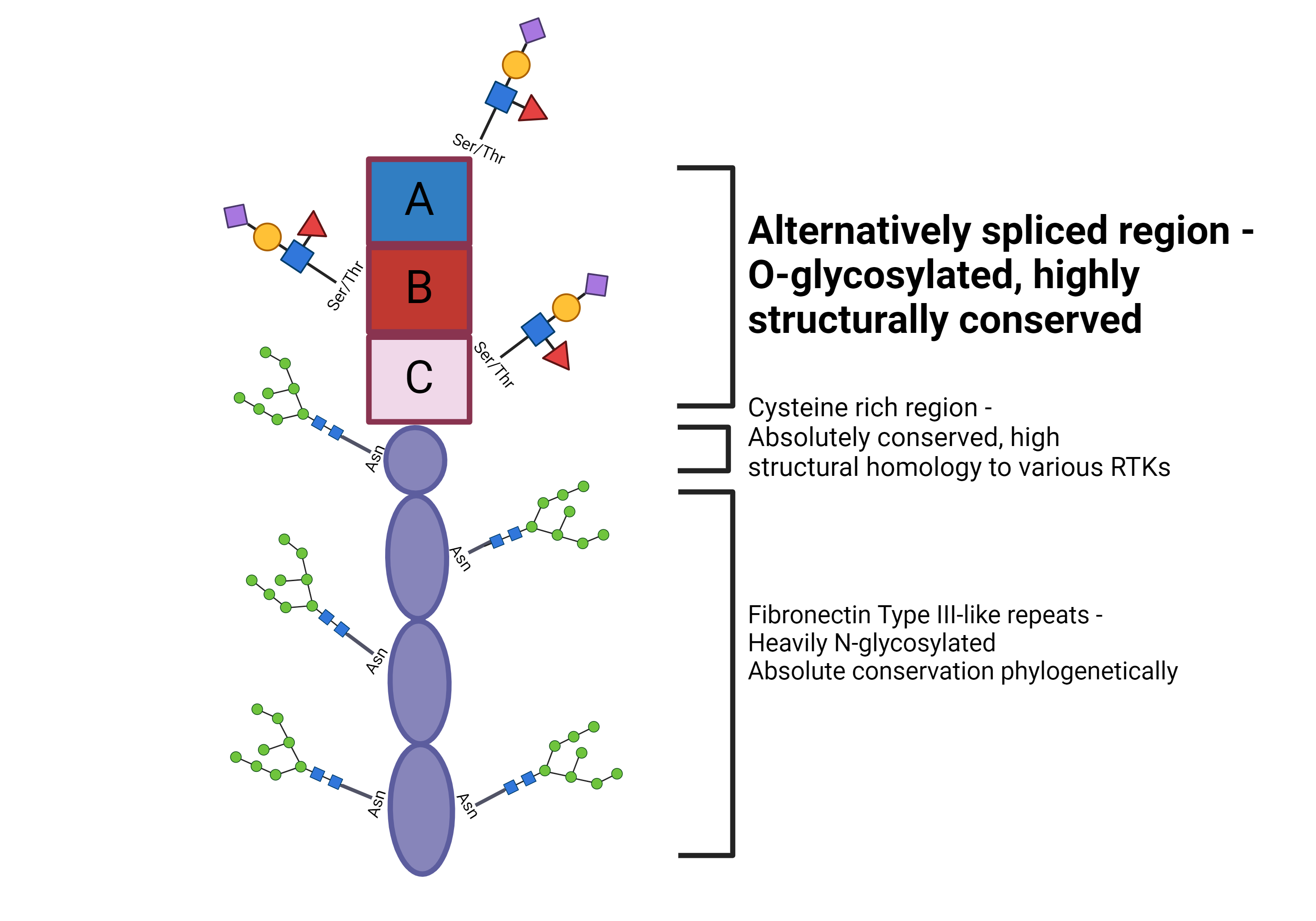


**Supplemental Figure 6. The CD45 ectodomain.** All isoforms of CD45 contain three distinct ectodomain regions: an alternatively spliced region (heavily O-glycosylated), a cysteine rich region, and three fibronectin type III-like repeats regions (heavily N-glycosylated). The alternatively spliced region shows structural conservation evolutionarily, and the cysteine-rich and fibronectin type-III repeats are absolutely conserved phylogenetically. The cytoplasmic domain (not shown) is also absolutely conserved and common to all isoforms.
